# Comparison of polystyrene nanoparticles and UV-inactivated antigen-displaying adenovirus for vaccine delivery in mice

**DOI:** 10.1186/1743-422X-10-108

**Published:** 2013-04-05

**Authors:** Lena Johrden, Matthias Tenbusch, Ruth Lietz, Michael Storcksdieck genannt Bonsmann, Thomas Niezold, Oliver Wildner, Wibke Bayer

**Affiliations:** 1 Department of Molecular and Medical Virology, Ruhr-University Bochum, Bochum, Germany; 2 Institute for Virology, University Hospital Essen, University Duisburg-Essen, Essen, Germany; 3 Current address: Paul-Ehrlich-Institute, Langen, Germany

**Keywords:** Nanoparticles, Polystyrene, Adenovirus, Ovalbumin, Vaccination

## Abstract

**Background:**

Inert nanoparticles are attracting attention as carriers for protein-based vaccines. Here we evaluate the immunogenicity of the model antigen ovalbumin delivered on polystyrene particles and directly compare particulate delivery with adenovirus-based immunization.

**Findings:**

Mice were vaccinated with soluble ovalbumin, ovalbumin-coated polystyrene particles of different sizes, or an adenovirus-based expression-display vector that encodes and displays a pIX-ovalbumin fusion protein. Antibody responses were clearly higher when ovalbumin was administered on polystyrene particles compared to soluble protein administration, regardless of the particle size. Compared to adenovirus-based immunization, antibody levels were lower if an equivalent amount of protein was delivered, and no cellular immune response was detectable.

**Conclusions:**

We demonstrate in a side-by-side comparison that inert nanoparticles allow for the reduction of the administered antigen amount compared to immunization with soluble protein and induce strongly enhanced antibody responses, but responses are lower compared to adenovirus-based immunization.

## Main text

Protein and peptide vaccines are generally regarded as safe; however, in most cases they require the co-administration of adjuvants to be effective, which may in turn cause adverse events [1]. Therefore it is desirable to develop safe and clinically effective non-adjuvanted vaccines, and one approach is the use of nano- or microparticles coated with antigen, which can enhance immunogenicity compared to soluble antigen [2-4]. In this study we analyzed polystyrene particles coated with the model antigen ovalbumin for their potency at inducing humoral and cellular responses. As adenoviral (Ad) vectors are very popular for vaccine development because of their high immunogenicity and strong immune responses to the delivered vaccine antigens, we made side-by-side comparisons of coated polystyrene particles of varying sizes with an Ad expression-display vector, which we showed before to induce especially good CD4^+^ T cell and antibody responses [5].

Carboxyl-modified polystyrene beads of 24, 60, 93, 220 and 340 nm in diameter (BangsLabs/Polysciences, Fishers, IN) were loaded with recombinant ovalbumin (Sigma, Munich, Germany) by the carbodiimide method [6], and antigen loading was verified by dot blot and enzyme-linked immunosorbent assay (ELISA; data not shown). For immunization experiments, 8–9 weeks old C57BL/6 mice (Élevage Janvier, Le Genest St Isle, France) were used. Mice were treated in accordance with the regulations and guidelines of the Institutional Animal Care and Use Committee of the Ruhr University Bochum, Germany.

**Figure 1:**
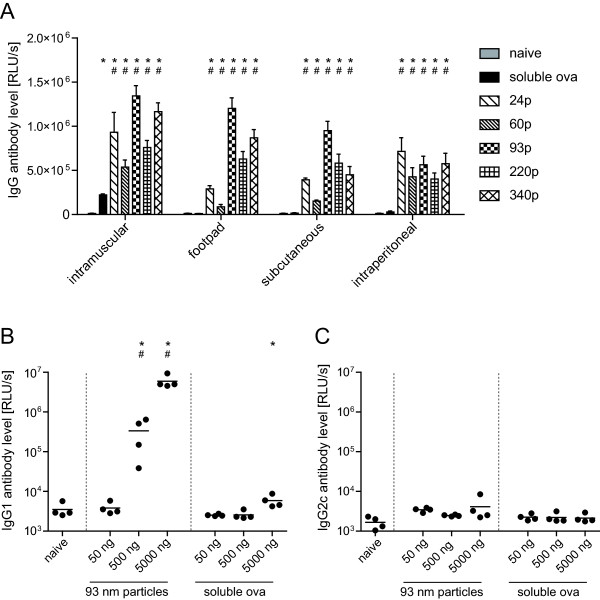
**Immune response after immunization with ovalbumin loaded polystyrene particles.** C57BL/6 mice were immunized once with 5 μg soluble ovalbumin (ova), or ovalbumin-coated polystyrene particles of 24 nm (24p), 60 nm (60p), 93 nm (93p), 220 nm (220p), or 340 nm diameter (340p) carrying 5 μg ovalbumin, either by intramuscular, footpad, subcutaneous or intraperitoneal injection. Two weeks after immunization ovalbumin specific IgG levels were determined luminometrically from 1:10000 dilutions of plasma by ELISA (**A**). To compare different amounts of protein, mice were immunized once by intramuscular injection with 50 ng, 500 ng, or 5000 ng of ovalbumin, either as soluble protein or coated on 93 nm polystyrene particles, and IgG1 (**B**) and IgG2c antibody titers (**C**) were analyzed in 1:100 dilution of plasma two weeks after immunization. Statistically significant differences compared to naïve mice or mice immunized with the respective amount of soluble ovalbumin are indicated by * or #, respectively (*P *< 0.05, Student-Newman-Keuls method). The graphs show mean values of RLU/s (relative light units per second) and standard errors of the means of 4 (**B**, **C**) or 8 mice per group (A); data was acquired in two independent experiments.

To determine the best route of administration for particle-based immunization, mice received 5 μg of soluble ovalbumin or ovalbumin loaded on polystyrene particles diluted in 100 μl sterile PBS by intramuscular, subcutaneous, or intraperitoneal injection, or by intradermal injection into both hind footpads. Ovalbumin-specific antibody levels were determined two weeks after immunization by ELISA [7] using ECL substrate (Alpha Innotech, San Leandro, USA) for luminometric readout. Soluble ovalbumin only induced a significant antibody level after immunization by intramuscular injection, but not the other routes, whereas all particles induced ovalbumin-binding antibodies regardless of the immunization route (Figure 1A). Antibody levels induced by ovalbumin-loaded particles were significantly higher than after immunization with soluble ovalbumin (Figure 1A). Next, we analyzed the amount of protein necessary for the induction of detectable antibody levels. For this, mice were immunized once by intramuscular injection with 50 ng, 500 ng, or 5 μg of ovalbumin, either as soluble protein or coupled to 93 nm polystyrene particles. While 5 μg of soluble ovalbumin were necessary to induce measurable IgG1 antibody titers, 500 ng on 93 nm particles were sufficient to induce high levels of IgG1 (Figure 1B). Antibodies of IgG2c subtype were not induced in detectable amounts by either vaccination strategy (Figure 1C).

To analyze whether the induction of antibodies by protein-loaded polystyrene particles is dependent on CD4^+^ T cell help, mice were immunized twice with 93 nm polystyrene particles delivering the intermediate amount of 500 ng ovalbumin, and depleted of CD4^+^ T cells around both time points of immunization by intraperitoneal injection of a CD4 antibody [8]. After mice were depleted of CD4^+^ cells during vaccination, no IgG1 antibodies were detectable (data not shown), suggesting the necessity of CD4^+^ T cell help for antibody induction.

Adenoviral vectors are very popular tools for vaccine development and they are known to be highly immunogenic [9]. We compared immune responses induced by polystyrene particles loaded with ovalbumin with an adenoviral ovalbumin expression-display vector; this Ad5 vector Ad.pIXova encodes a fusion protein of the capsid protein IX and ovalbumin, and hence displays ovalbumin on the particle [5]. The ovalbumin content of Ad.pIXova was determined by ELISA to be 50 ng per 10^10^ viral particles (vp; data not shown). To include a control for the influence of *in vivo* expression of ovalbumin from the adenoviral vector, a UV-inactivated [10] Ad.pIXova (UV-Ad^pIXova^) was included. Mice were immunized twice in a three-week interval by intradermal injection into the hind footpads with 50 ng soluble ovalbumin, 50 ng ovalbumin coupled to 24 nm, 93 nm, or 340 nm polystyrene particles, or 10^10^ vp Ad.pIXova or UV-Ad^pIXova^, equaling a protein delivery of 50 ng ovalbumin; immune responses were analyzed three weeks after the second immunization.

**Figure 2:**
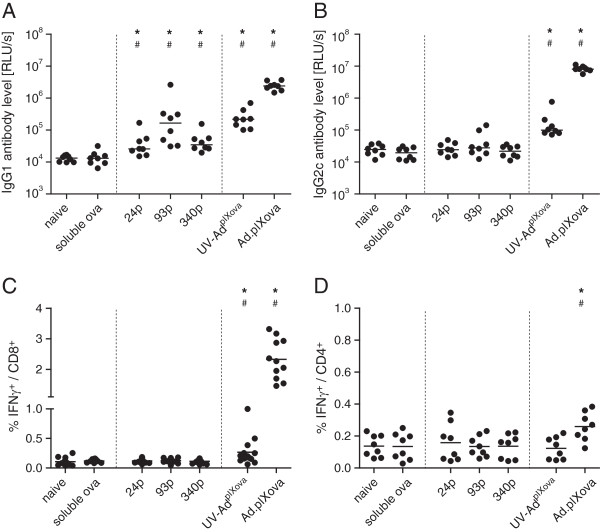
**Immune response after immunization with ovalbumin loaded polystyrene particles or adenoviral vectors.** C57BL/6 mice were immunized twice by intradermal injection into the hind footpads with 50 ng soluble ovalbumin, ovalbumin-coated polystyrene particles of 24 nm (24p), 93 nm (93p) or 340 nm diameter (340p) carrying 50 ng ovalbumin, or 10^10 ^vp ovalbumin-displaying adenovirus Ad.pIXova or UV-Ad^pIXova^. Three weeks after boost-immunization, ovalbumin binding IgG1 (**A**) and IgG2c antibody levels were analyzed from 1:100 dilutions of plasma (**B**). For analysis of cellular immune responses, spleen cells were isolated and restimulated *in vitro *with ovalbumin-specific peptides, and IFNγ expression by CD8^+ ^(**C**) and CD4^+ ^T cells (**D**) was analyzed by flow cytometry. Statistically significant differences compared to naïve mice or mice immunized with soluble ovalbumin are indicated by * or #, respectively (*P *< 0.05, Student-Newman-Keuls method). The graphs show data of 8 mice per group that was acquired in two independent experiments, the bars indicate mean values.

Immunization of mice with active or inactivated adenoviral vectors resulted in detectable levels of both IgG1 and IgG2c type ovalbumin-binding antibodies (Figure 2A and B). While the immunization with 50 ng soluble ovalbumin did not induce detectable antibodies of either subtype, mice immunized with polystyrene particles carrying the same amount of ovalbumin exhibited binding antibody responses of mainly IgG1 type, with the highest response in mice immunized with 93 nm particles, which showed levels similar to mice immunized with UV-Ad^pIXova^.

For the analysis of cellular immune responses, splenocytes were isolated and restimulated *in vitro* with ovalbumin-derived peptides [11,12], and flow-cytometric analysis of intracellular cytokine levels was performed. The numbers of IFNγ producing CD8^+^ (Figure 2C) and CD4^+^ T cells (Figure 2D) were highest in mice immunized with Ad.pIXova, and significantly lower when mice were immunized with the UV-inactivated UV-Ad^pIXova^ or polystyrene particles carrying 50 ng ovalbumin. While there is a tendency to higher CD8^+^ T cell levels in mice immunized with UV-Ad^pIXova^ than polystyrene particles, the levels are comparable in these groups and underline the importance of antigen expression by the Ad vector for strong CTL induction, as well as for induction of CD4^+^ T cell responses, as was described before [5]. A similar trend was observed when the concentration of IL4 was analyzed in the supernatant of restimulated splenocytes, although the levels were rather low in all groups (data not shown).

**Figure 3:**
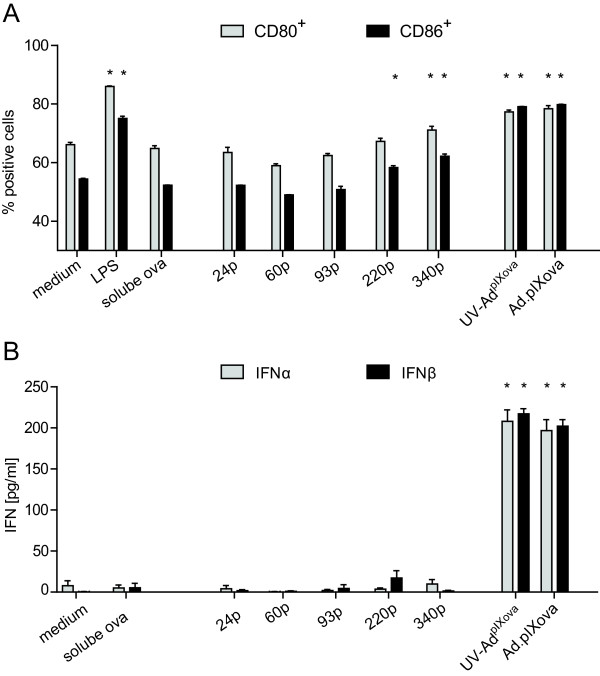
**Activation of dendritic cells by ovalbumin-loaded particles. **5×10^5^ bone-marrow derived DCs were co-incubated with 10 ng soluble ovalbumin, polystyrene particles loaded with 10 ng ovalbumin, or with 10^9 ^vp Ad.pIXova or UV-Ad^pIXova^. Incubation with medium or lipopolysaccharide (LPS) was used as negative and positive controls, respectively. After 16 h co-incubation, cells were stained for expression of the activation markers CD80 and CD86 and analyzed by flow cytometry (**A**). Supernatants of the cells were analyzed by ELISA for interferon α and β concentrations (**B**). Statistically significant differences compared to untreated DCs are indicated by * (*P *< 0.05, Student-Newman-Keuls method).

As no relevant cellular immunity was induced by polystyrene particle immunization, we wanted to further assess the immunogenicity of the particles, and analyzed activation of and interferon production by dendritic cells (DCs) after uptake of ovalbumin-loaded particles or adenoviral vectors. Some low degree of activation of DCs was observed after loading with the largest, 220 nm and 340 nm polystyrene particles, whereas incubation with adenoviral particles led to DC activation comparable to stimulation with LPS (Figure 3A). Adenoviral particles also induced significant levels of interferon α and interferon β, but DCs incubated with soluble ovalbumin or ovalbumin-coated polystyrene particles did not produce interferons (Figure 3B). These findings confirm the inert nature of the polystyrene particles.

Comparing the immune responses induced by adenovirus-based immunization with immune responses induced by polystyrene particles loaded with a high amount of protein, i.e. 5 μg, it can be observed that the antibody levels that can be reached by immunization with protein-coated 93 nm particles are actually comparable in strength. It has been reported before that the particle size determines the induced immune response (reviewed in [13]), and our findings confirm this, as the best antibody response was found for the 93 nm particles, while only the smallest, 24 nm particles, that can most easily enter lymphatic structures on their own [14], could induce a significant, albeit low, CD8^+^ T cell response (data not shown).

Our results indicate that antigen coupling to polystyrene particles, although they are immunologically inert, induces enhanced antibody responses in comparison to immunization with soluble protein, and thus allows for the reduction of protein amount used for protein-based immunization. In contrast to adenoviral vectors, delivery on polystyrene particles does not change the antibody profile compared to protein immunization, which consists predominantly of IgG1 subtype antibodies.

We confirmed that polystyrene particles themselves are not immunogenic, having no particular effect on DC activation and cytokine production, thus they should be associated with very little risk of inducing autoimmunity as recently described for other vaccine adjuvants [15] (and reviewed in [16]). Rather, the enhancing effect of polystyrene particle delivery seems to rely mainly on the particulate nature and the size of the particles, as well as on the presentation of the antigen in an ordered array. Polystyrene particles are inert but not degradable, however, biodegradable particles such as dextran particles should be comparably adequate protein carriers and appropriate for use in humans. While the cellular immune response to adenoviral vectors was much higher than to antigen-coated polystyrene particles, in cases where protection can be conferred by humoral immunity, or for protein delivery in prime-boost combinations with viral vectors, particle-mediated delivery is an attractive option to efficiently and safely enhance immune responses to protein-based vaccines.

## Competing interest

The authors declare that they have no conflict of interest.

## Authors’ contributions

LJ produced the vaccines and carried out the animal experiments and analyses. MT participated in the study design, data analysis and the drafting of the manuscript. RL, MS and TN participated in the animal experiments. OW designed the study and participated in the drafting of this manuscript. WB participated in the study design, data analysis, and drafted the manuscript. All authors read and approved the final manuscript.
